# Statistical Optimization of Culture Conditions for Enhanced Biomass Yield of *Lactobacillus acidophilus* CM1 Using Plackett–Burman and Response Surface Methodology

**DOI:** 10.1155/ijfo/5576637

**Published:** 2025-06-19

**Authors:** Khushboo Rajput, Amar Yasser Jassim, Ameer Abbas Mohammed, Abdel Rahman Mohammad Said Al-Tawaha, Arun Karnwal, Tabarak Malik

**Affiliations:** ^1^Department of Microbiology, School of Bioengineering and Biosciences, Lovely Professional University, Phagwara, Punjab, India; ^2^Department of Marine Vertebrate, Marine Science Center, University of Basrah, Basrah, Iraq; ^3^Department of Biological Sciences, Al Hussein Bin Talal University Ma'an, Ma'an, Jordan; ^4^Department of Microbiology, Graphic Era (Deemed to Be University), Dehradun, Uttarakhand, India; ^5^Department of Biomedical Sciences, Institute of Health, Jimma University, Jimma, Ethiopia

**Keywords:** food security, healthcare, low-cost medium, optimize growth media, Placket–Burman design, public health

## Abstract

This study is aimed at enhancing the biomass yield of *Lactobacillus acidophilus* CM1 by identifying and optimizing critical growth parameters. Using the Plackett–Burman design (PBD), 11 physical and chemical variables were screened, of which pH, temperature, NaCl concentration, and inoculum size were found to significantly influence cell growth (*p* < 0.05). These statistically significant factors were subsequently optimized using response surface methodology (RSM) with a central composite design (CCD). Optimization led to a 1.45-fold increase in biomass yield, achieving a maximum of 1.948 g/100 mL. ANOVA confirmed model validity with an *R*^2^ of 0.9689 and adequate precision of 52.77, indicating a strong predictive capability. The integration of PBD and RSM-CCD proved efficient for minimizing experimental runs while maximizing output, supporting the development of cost-effective cultivation strategies for probiotic production. This approach offers a scalable model for bioprocess optimization in industrial fermentation.

## 1. Introduction

*Lactobacillus* species are among the most widely employed probiotic bacteria in the food and pharmaceutical sectors, celebrated for their health-promoting attributes and extensive applications [1]. In medicinal contexts, these lactic acid bacteria (LAB) are precious as they support gut microbiota colonization, which is beneficial in managing gastrointestinal disorders, liver conditions, postantibiotic recovery, and microbial imbalances [2]. The broad health benefits associated with these probiotics have driven substantial scientific interest in their physiological properties and development requirements. The cultivation of *Lactobacilli*, however, presents specific nutritional demands that require a nutrient-dense medium for optimal growth [3]. Among available media, Man–Rogosa–Sharpe (MRS) medium is widely recognized as the standard for LAB cultivation due to its ability to support robust bacterial growth [4, 5]. Nonetheless, commercial probiotic production often incurs high costs due to the expense of specialized media and the complexity of manufacturing processes, a challenge that has spurred ongoing research into optimizing cost-effective growth environments for these bacteria [6, 7].

Research efforts have frequently targeted medium optimization to enhance growth conditions for specific *Lactobacillus* strains. For instance, studies on the dairy-associated *L. rhamnosus* strain have examined the effects of varying carbon and nitrogen sources, essential growth factors (including amino acids and vitamins), and culture conditions (such as temperature, pH, and oxygen availability) on bacterial proliferation. For example, researchers [8] demonstrated that a medium containing yeast extract (6.0% w/v), glucose (5.01% w/v), a vitamin solution (1.28 w/v), and an adjusted pH of 6.9 yielded higher viable cell counts than the traditional MRS medium, illustrating the impact of nutrient-specific modifications. Similar optimization studies have investigated growth conditions for *Lactobacillus acidophilus* CM1 [9], a strain derived from various fermented and nonfermented food sources, to identify cost-effective, high-yield media formulations.

To identify optimal medium compositions efficiently, Plackett–Burman design (PBD) has proven instrumental, offering a systematic approach to screen multiple factors with minimal experimental runs. This design is particularly advantageous in resource-limited or time-sensitive scenarios, allowing researchers to rapidly identify critical growth factors [10, 11].

The PBD is a two-level factorial design that evaluates experimental factors at predefined high and low levels based on prior data or practical considerations. It maintains orthogonality, ensuring statistical independence among variables and accurate estimation of main effects without confounding interactions. As a fractional design, PBD tests only a subset of all possible combinations, making it efficient for screening numerous variables. The design's resolution determines how many factors can be tested without overlap—Resolution III screens up to N−1N-1N−1 factors, while Resolution IV screens up to N−2N-2N−2. Randomization of runs helps reduce bias from external influences. PBD is especially valuable in bioprocess optimization, where it identifies key growth factors like pH or temperature, streamlining the transition to more detailed optimization methods such as response surface methodology (RSM). Unlike one-factor-at-a-time approaches, which are time-consuming and ignore interactions, PBD provides a rapid and scalable method to enhance microbial yield by refining media and process conditions.

In this context, growth medium optimization generally follows two phases: initial screening to identify critical ingredients and refinement based on Pareto's principle [12, 13]. With the PBD, researchers can examine \(*n*\) variables across \(*n* + 1\) experimental runs, minimizing resource consumption while retaining the design's orthogonality to prevent confounding between variables [7, 14, 15]. This study is aimed at enhancing the growth of a probiotic strain in a medium formulated with variable carbon and nitrogen sources. A secondary is aimed at evaluating the effects of added mineral salts and growth factors to improve bacterial growth further. Ultimately, this research seeks to identify a cost-effective medium composition that supports high biomass production of *L. acidophilus* CM1, advancing its commercial viability as a probiotic supplement.

## 2. Materials and Methodology

In this study, *Lactobacillus acidophilus* CM1, isolated from cow milk as previously reported [9, 16], was cultured and maintained on MRS medium using standard plating techniques. Identification of the strain was confirmed via biochemical assays and 16S rRNA sequencing (submitted to Genbank with ID OP811266.1), ensuring precise classification within the laboratory setting (Khushnoo and Karnwal, 2023). The bacterial isolates were preserved in glycerol stocks for subsequent analyses to retain viability over extended storage periods.

### 2.1. Inoculum Preparation

The inoculum of *Lactobacillus acidophilus* CM1 was prepared by harvesting actively growing colonies from MRS agar plates into sterile physiological saline (0.85% NaCl) or MRS broth. The bacterial suspension was adjusted to match a 0.5 McFarland standard, corresponding to an approximate concentration of 1.5 × 10^8^ CFU/mL. The standardized inoculum was then used for all experimental runs to ensure reproducibility and accuracy, particularly in statistical optimization studies employing PBD and RSM. Uniform inoculum concentration was essential for assessing the true impact of physicochemical parameters on bacterial growth and biomass yield.

### 2.2. LAB Growth Medium

A growth medium formulated explicitly for LAB was developed, incorporating a variety of components that include protease peptone, beef extract, yeast extract, dextrose, Polysorbate 80, ammonium citrate, sodium acetate, magnesium sulfate, manganese sulfate, and dipotassium phosphate. The production process was carried out under carefully regulated conditions, with the initial pH set to 7.0 and the temperature maintained at 30°C. This environment was sustained for 24–48 h, facilitating optimal growth and proliferation of LAB.

### 2.3. Medium Preparation

The medium used for cultivating *Lactobacillus acidophilus* CM1 was specifically formulated to support the growth of LAB. The base composition included protease peptone, beef extract, yeast extract, dextrose, Polysorbate 80, ammonium citrate, sodium acetate, magnesium sulfate, manganese sulfate, and dipotassium phosphate. The pH of the medium was initially adjusted to 7.0 and sterilized before inoculation. Cultivation conditions such as temperature and incubation time were optimized based on statistical design models to maximize cell biomass (Khushboo and Karnwal, 2023). The prepared medium provided essential nutrients and optimal physical–chemical conditions required for robust microbial proliferation and was key in achieving a 1.45-fold increase in biomass yield under optimized parameters.

### 2.4. Approach Used for Response Determination

To determine the response in this study, we used a systematic statistical approach combining PBD and RSM. Initially, PBD was employed to screen and identify key factors—such as pH, temperature, NaCl concentration, and inoculum size—that significantly influenced the biomass yield of *Lactobacillus acidophilus* CM1. Once these critical variables were pinpointed, RSM, particularly a central composite design (CCD), was used to optimize their levels to maximize cell growth. The model's effectiveness was validated using analysis of variance (ANOVA), regression analysis, and statistical metrics such as *R*^2^ and adeq precision, ensuring the reliability of the predicted responses. Thus, the response—measured as biomass yield in g/100 mL—was determined by analyzing how changes in these key variables affected the output under controlled experimental conditions.

### 2.5. Screening of Physical and Chemical Parameters Using PBD

Statistical experimental design (SED), commonly called the design of experiments (DOE), represents a systematic methodology to optimize data output while minimizing the number of analyses required. In contexts characterized by numerous potential causal factors that may influence one or more responses, a critical technique known as “screening design” is employed to identify the most significant components affecting the outcome. This approach simplifies subsequent trials by evaluating a reduced set of variables. Prior screening studies are essential in eliminating irrelevant factors and conserving the time and resources required for further research.

Among the various methodologies available for screening, Plackett–Burman (P.B.) orthogonal arrays, developed by Plackett and Burman, are particularly advantageous. These designs enable accurate estimation of the direct impacts of individual factors within the experimental framework. When screening “*n*” components, the PBD technique utilizes *n* + 1 runs, maintaining the sample size as a multiple of four, denoted as 4^k observations, where *k* represents a number ranging from 1 to *n*. This distinctive characteristic defines the PBD, rendering it especially suitable for *n* × 4 configurations (such as 8, 12, 16, and 20) in experiments involving more than seven variables (e.g., 7, 11, 15, and 19).

To effectively implement a two-level factorial design, three critical requirements must be satisfied:

1. Factor selection: Factors should be judiciously chosen to yield optimal responses while remaining computationally manageable.
2. Range selection: The selected ranges for each factor must be adequate to compute their respective effects accurately.
3. Avoidance of overlapping ranges: The ranges established for each factor should not overlap with those of other factors to avert complications in experimental setup and factor interaction failures.

In the current study, LAB biomass production, influenced by 11 distinct factors, was evaluated utilizing the PBD, with a relative significance threshold set at 95% for the experimental trials. The basic values of independent variables were coded using the P.B. design into high (+1) and low (−1) levels, as illustrated in Table 1. This methodological framework allows for a robust analysis of the factors influencing LAB biomass production, ultimately leading to more efficient and targeted experimental outcomes.

## 3. Result and Discussion

DOE is a systematic mathematical method employed to rigorously manage, operate, and evaluate trials to discover how input variables (independent variables) affect output responses (dependent variables). This method helps pinpoint key factors that impact outcomes and determines the best conditions for each, providing a clear structure for improving processes and systems.

The core principle of the DOE is carefully planning and conducting experiments where variables are deliberately adjusted, and the resulting outcomes are measured under regulated conditions. This systematic approach enables researchers to discern the most influential factors affecting the response and quantify each variable's impact [17]. Furthermore, DOE can elucidate interactions between these factors and facilitate the identification of optimal settings for input variables that yield desired output responses [18]. The applications of DOE are extensive, encompassing various industries, including manufacturing, engineering, and pharmaceuticals, where it is leveraged to improve product quality, enhance process efficiency, and achieve cost savings.

The DOE process comprises several key steps: defining the problem and objectives of the experiment, selecting relevant variables and their respective ranges, choosing an appropriate experimental design, executing the experiments, collecting data, conducting statistical analyses, and formulating conclusions and recommendations based on the experimental findings. By pinpointing the critical elements that influence a process or system, DOE supports significant enhancements in quality, efficiency, and cost-effectiveness, thus guiding decision-making with empirical evidence [19].

In this study, we employed Design Expert 12.0 software to conduct 12 experimental runs (detailed in Table 2) to identify the essential factors influencing microbial growth. The main objective of this screening analysis was to evaluate the effects of 11 factors on cell growth, quantified in g/100 mL. The variables under investigation included pH, temperature, NaCl concentration, bile salt level, inoculum percentage, incubation period, ascorbic acid content, ammonium citrate level, magnesium sulfate concentration, manganese sulfate concentration heptahydrate concentration, and calcium carbonate concentration. Twelve flask tests, which included three center points, were conducted at a 95% significance level. The significance of the 11 variables was assessed using a Student's *t*-test, with the experimental and predicted cell growth values presented in Table 3.

**Figure 1:**
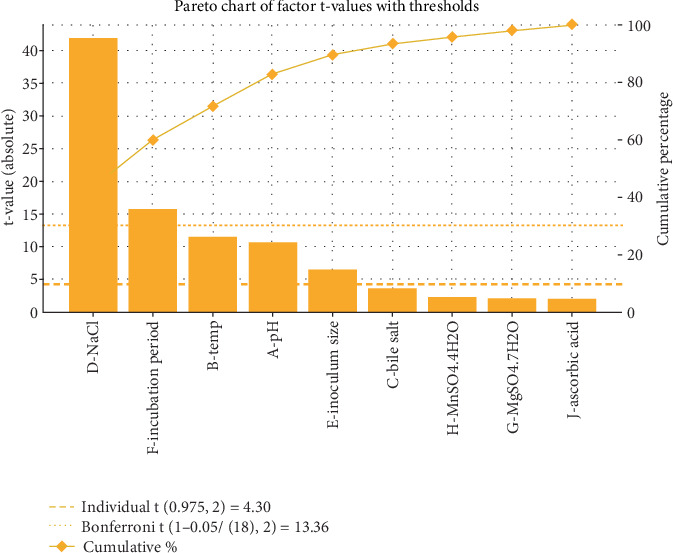
Pareto chart of standardized effects of growth parameters on *Lactobacillus acidophilus* CM1 growth: identification of significant factors (NaCl, pH, temperature, and inoculum size) based on *t*-value threshold.

Statistical analysis of cell growth (g/100 mL) for the PBD is displayed as an ANOVA table in Table 4. Furthermore, Figure 1 shows a Pareto chart illustrating the impact of variables on cell growth.

The Model *F*-value of 257.42 indicates that the model is statistically significant, with a mere 0.39% probability that an *F*-value of this magnitude could arise from random noise. Furthermore, the analysis of *p* values reveals that Terms A, B, D, E, and F are statistically significant, as their *p* values are less than the conventional threshold of 0.0500. In contrast, terms with *p* values exceeding 0.1000 suggest a lack of significance. In instances where numerous model terms are deemed insignificant—excluding those necessary to uphold the hierarchical structure—considering model reduction may enhance the overall performance and interpretability of the model (B—temperature (*p* value = 0.0075), A—pH (*p* value = 0.0087), D—NaCl (*p* value = 0.0006), and F—incubation period (*p* value = 0.004)).

### 3.1. Pareto Chart

A Pareto chart is an effective visual tool for demonstrating the proportional relevance of different factors in a PBD. This chart methodically organizes variables according to their impact on the response variable, systematically ranking them from most to least influential [20]. As depicted in Figure 1, the Pareto chart analyzes nine variables that affect maximum cell growth. Notably, four variables—NaCl concentration, pH, temperature, and inoculum size—surpassed the critical threshold t-value of 4.30, establishing their significant role in enhancing cell growth. The height of each bar in the chart indicates the degree of influence that these variables have.

By employing the Pareto chart, researchers can prioritize areas for further investigation or optimization, directing focus toward the most critical factors impacting the response variable. Additionally, this visualization fosters clear and concise communication of the findings derived from the P.B. design, enabling stakeholders to readily comprehend the key determinants influencing the experimental outcomes.

### 3.2. Predicted Vs. Actual Values

The comparison between projected and actual data, as illustrated in the projected versus actual plot, provides a robust method for evaluating the validity and accuracy of the PBD model [16, 21, 22]. The PBDmodel generates anticipated values in this context, while the experimental data represents the observed values. Ideally, the data points within this plot should align along a straight line with a slope of 1.0, signifying that the P.B. design model is both accurate and valid.

**Figure 2:**
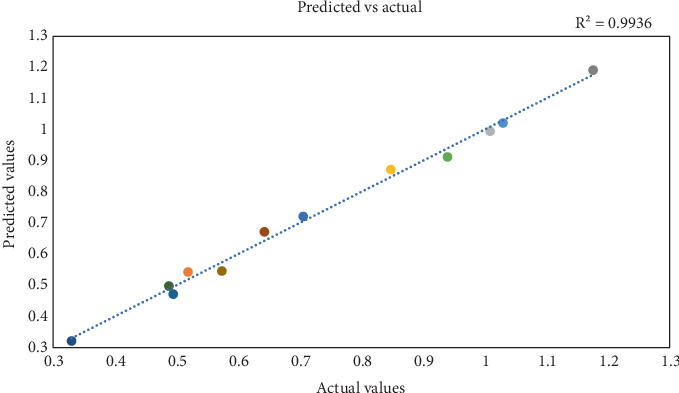
The predicted vs. actual bacterial cell yield of PBD for 11 factors (12 runs).

As shown in Figure 2, the data derived from 11 experimental runs, encompassing 11 variables, closely adheres to this straight line, indicating a high degree of reliability and a lack of outliers. Any deviations from this expected linear relationship may suggest the potential invalidity of the PBD model or the influence of additional factors on the response variable that was not previously considered.

Furthermore, the anticipated versus actual plot serves as a valuable tool for identifying outliers or other data patterns that could undermine the reliability of the PBD model. Outliers, defined by substantial deviations from the overall data trend, may indicate experimental errors or unique conditions during the study. By meticulously analyzing the projected versus actual plot, researchers can critically assess the accuracy and dependability of the PBD model, determining whether any adjustments or modifications are warranted to enhance its predictive capability [23].

### 3.3. Box–Cox Transformation

The Box–Cox transformation is a popular statistical method for modifying datasets to meet the uniformity and uniform variance requirements, which are needed for many statistical analyses. This transformation adds a power function to the data, which helps lessen the impact of anomalies and other causes of variability. When integrated with perturbation in a PBD, the Box–Cox transformation and perturbation can improve the consistency and strength of the results [22, 25].

**Figure 3:**
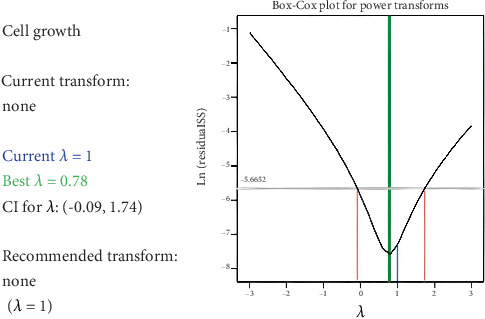
Box–Cox plot of P.B. design for 11 factors, showing data normalization without the need for transformation [24].

The process begins with implementing a PBD, where the initial experimental setup is conducted [24]. If necessary, researchers may perturb the system by adjusting the factor levels, as previously outlined. However, it is crucial to note that if the response variable fails to conform to a normal distribution or exhibits heteroscedasticity, the integrity of the PBD outcomes may be compromised. In such cases, the Box–Cox transformation on the response variable can help address these challenges. This transformation is aimed at standardizing the data and lessen anomalies' influence. However, as shown in Figure 3, the Box–Cox plot indicates no transformation is needed here. The data from the PBD, which includes 11 factors, is adequately normalized, confirming that the assumptions of normality and equal variance are satisfied without any additional modifications.

Regression formula for the model's cell growth g/100 mL

 $$\begin{array}{c} 0.935019+\left(0.037556\times pH\right)+\left(0.006100\times Temperature\right)+\left(0.025556\times Bile\;Salt\right)-\left(0.073889\times NaCl\right)-\left(0.027600\times Inoculum\;size\right)–\left(0.002329\times Incubation\;period\right)+\left(0.011754\times MgSO4.7H2O\right)+\left(0.012456\times MnSO4.4H2O\right)–\left(0.011404\times Ascorbic\;acid\right). \end{array}$$

### 3.4. Optimization of Microbial Cell Growth Factors

The main goal of this experimental screening design was to identify the key variables affecting microbial cell proliferation. PBD studies were employed as an initial approach to identify potentially influential production parameters. This systematic investigation is aimed at discerning key factors before advancing to a more focused screening process. The selection of experimental variables was guided by their previously observed correlations with production outcomes in prior shake flask cultures.

A Pareto chart provided a complete overview of the data, visualizing the typical impact of all variables on microbial productivity. The investigation found that the amount of sodium chloride (NaCl), pH, temperature, and incubation length significantly impacted cell development [5, 6], with temperature and pH emerging as critical determinants for enhancing growth performance. While using the PBD has been a common strategy in numerous studies aimed at optimizing parameters for microbial cell proliferation, this study differentiates itself by preselecting factors before implementing PBD, a method necessitated by the observed variations among different microbial strains.

Previous research has extensively examined the influence of physical parameters, such as temperature and pH, on microbial growth [10, 26]. Furthermore, researchers [20] emphasized the importance of medium composition in optimizing cell growth. The factors identified through the PBD were subsequently optimized using RSM with a Box-Behnken experimental framework. Results were analyzed utilizing Design-Expert software, with the data subjected to rigorous ANOVA based on the established experimental design. The highest cell growth recorded in this study was 1.948 g/100 mL, and the close correlation between experimental and predicted data validates the model's efficacy, which was further assessed using multiple evaluative criteria.

Notably, the regression equation derived from quadratic regression analysis on the experimental data delineates the relationship between cell growth and the various optimizing parameters. Previous investigations have underscored the crucial role of medium composition in bacterial proliferation [27]. Although many studies have harnessed PBD for optimizing cell mass production, this research adopts a distinctive methodology by sequentially selecting factors before applying PBD. This strategic approach accounts for the variability of factors across different strains [26]. Moreover, recent initiatives have sought to minimize medium costs [4]. This study incorporated specific nitrogen sources, namely, peptone and yeast extract, into the MRS medium, alleviating production's financial burdens.

Earlier investigations by researchers [18] identified inoculum size and temperature as pivotal factors positively influencing microbial growth. The high *R*^2^ value of 0.9689, accompanied by an adjusted *R*^2^ value of 0.9953, reflects a robust agreement between the experimental and predicted levels of bacteriocin production [18]. This strong correlation suggests that the model proficiently predicts responses, particularly as the difference between the predicted *R*^2^ and adjusted *R*^2^ remains below the threshold of 0.2, further attesting to its reliability. Additionally, the adeq precision, which quantifies the signal-to-noise ratio, exceeds the recommended threshold of 4, registering a ratio of 52.777, indicating sufficient signal strength.

This model adeptly traverses the design space, facilitating evaluating of experimental factors relative to noise, as highlighted by researchers [19]. The model's *F*-value of 257.42, with a corresponding *p* value of 0.0039, underscores the significance of the model terms. Specifically, factors A, B, Interactions A.C. and B.C., and the squared terms *B*^2^ and *C*^2^ were determined to be significant contributors to cell growth, corroborating findings by researchers [12]. Detailed insights regarding the ANOVA analysis related to these findings are presented in Table 4.

### 3.5. RSM

This study employed a CCD model to optimize the medium components utilizing RSM as shown in Table 5 [16]. The four independent variables were designated as *X*1, *X*2, *X*3, and *X*4, with their respective concentrations ranging from 9.29 g/L to 32.6 g/L, 7.34 g/L to 54.7 g/L, 5.76 g/L to 62.8 g/L, and 8.14 g/L to 59.6 g/L. The optimal concentrations for these variables were systematically determined and are presented in Table 4, illustrating both coded units and actual values.

The design matrix comprised 20 experimental runs, each reflecting different combinations of the independent variables. The results of these experiments and the corresponding biomass production data are summarized in Table 4. The biomass production observed varied significantly, ranging from 1.094 to 1.948 g/L.

Notably, Run 19, characterized by a pH of 2.5, a temperature of 35°C, a NaCl concentration of 3 g/L, and an incubation period of 30 h, yielded the highest biomass production. This finding underscores the significance of the identified optimal conditions, as they facilitated the most advantageous outcome for biomass production throughout the experimentation process [7, 28].

### 3.6. Final Equation in Terms of Coded Factors

 
$$\begin{array}{c} Y=+1.36-0.0615\;A-0.0983\;B+0.0038\;C+0.1167\;D+0.0088\text{ AB}–0.022\text{ AC}-0.0181\text{ AD}–0.0152\text{ BC}–0.0034\text{ BD}=0.0134\text{ CD} \end{array}$$

The coded equation in terms of the factors allows for predictions regarding the response at specified levels of each factor. By convention, high factor levels are represented as +1, while low levels are denoted as −1. This coded representation facilitates the assessment of the factors' relative impacts by comparing their coefficients [11, 16].

The significance of the quadratic regression model, which incorporates linear, squared, and interaction terms, is substantiated by the ANOVA presented in Table 4 of this study. The model demonstrates a strong significance, as evidenced by an *F*-value of 33.22 and a very low probability value (*p* > *F*) of 0.0001. The *F*-value, the ratio of the mean square regression to the mean square residual, is a critical indicator of the model's validity. Given that the estimated *F*-value substantially exceeds this critical threshold, we reject the null hypothesis, thereby confirming the model's high significance in elucidating the relationships among the variables.

Furthermore, the model's goodness of fit is evaluated using the coefficient of determination (*R*^2^), which was calculated to be 0.9676. This indicates that the model accounts for approximately 96.76% of the total variability in the response variable, leaving only 3.24% unexplained. *R*^2^ values range from 0 to 1, with values approaching 1 signifying greater predictive accuracy. In this context, the model is deemed robust, as it effectively predicts the response and exhibits an *R*^2^ exceeding 0.75, affirming its appropriateness for the analysis [7].

The difference between the predicted *R*^2^ of 0.1897 and the adjusted *R*^2^ of 0.3377 is less than 0.2, indicating reasonable agreement between the two values (Table 6).

Adeq precision measures the signal-to-noise ratio. It is preferable to have a ratio that is bigger than 4. The ratio of 7.0546 demonstrates that the signal is sufficient. Using this paradigm, one may move more easily through the design space.

### 3.7. ANOVA for 2FIT Model

Factor coding entails employing coding techniques to represent categorical variables numerically. This transformation is crucial for statistical analysis, allowing categorical data to be integrated into quantitative models. The sum of squares is a fundamental mathematical calculation that involves summing the squares of a given set of numbers, serving as a measure of variability within the data.

Within statistical analysis, Type III partial rewriting refers to converting text into an academic style while ensuring no additional information is introduced. The primary objective of this approach is to enhance clarity and coherence in the presentation of findings.

This study's calculated Model *F*-value of 2.48 indicates the model's statistical significance (Table 7). The likelihood of obtaining an *F*-value of this magnitude purely due to random variation is estimated at 4.25%. This low probability suggests that the observed relationships are unlikely to have occurred by chance.

*p* values below the threshold of 0.0500 signify that the model terms exhibit statistical significance. Specifically, Model Terms B and D demonstrate considerable significance in the current analysis. Conversely, values exceeding 0.1000 indicate a lack of statistical significance among those model terms. Implementing model reduction strategies may enhance the overall quality of the model, particularly if a substantial portion of the associated terms is deemed superfluous, aside from those necessary to maintain the hierarchical structure of the model.

The *F*-value of 0.70 for the lack of fit reveals that the lack of fit is not statistically significant compared to the pure error. The estimated probability of observing a lack-of-fit *F*-value of this magnitude due solely to random variation is approximately 72.69%. A nonsignificant lack of fit is favorable, suggesting that the model adequately captures the underlying data structure.

## 4. Conclusion

This study successfully employed a two-step statistical approach—PBD followed by RSM—to optimize the cultivation conditions of *Lactobacillus acidophilus* CM1, thereby enhancing its biomass yield while aiming to reduce overall production costs. The screening phase using PBD identified pH, temperature, NaCl concentration, and inoculum size as the most influential factors affecting cell growth. These variables were subsequently optimized using a CCD under RSM, leading to a 1.45-fold improvement in biomass yield compared to unoptimized conditions.

The maximum biomass production achieved was 1.948 g/100 mL under the optimized conditions: pH of 2.5, temperature of 35°C, NaCl concentration of 3%, and an incubation period of 30 h. These findings underline the significance of maintaining slightly acidic conditions and moderate salinity alongside a carefully timed incubation period to promote optimal growth of *L. acidophilus* CM1. Statistical analysis validated the robustness of the optimization model, with an *R*^2^ of 0.9689, adjusted *R*^2^ of 0.9953, and a high adequate precision value of 52.77, reflecting the model's reliability and predictive accuracy.

The integration of PBD and RSM offered a comprehensive and cost-effective framework for bioprocess optimization, significantly reducing the number of experimental runs required to identify and fine-tune critical growth parameters. Moreover, using standard but economical nitrogen sources such as yeast extract and peptone in a modified MRS medium further supports the feasibility of scaling up this process in industrial settings. This work provides a solid foundation for commercializing *L. acidophilus* CM1 as a probiotic in food and pharmaceutical applications. Future work can extend these optimization strategies to alternative substrates, scale-up trials in bioreactors, and exploration of cocultivation models with other probiotic strains. The study advances microbial bioprocess optimization by demonstrating how factorial experimental designs can systematically improve yield and cost efficiency, aligning with the growing global demand for affordable and high-quality probiotic formulations.

## Figures and Tables

**Table 1:** A Plackett–Burman design was employed to identify and optimize the different experimental factors influencing the growth of *Lactobacillus acidophilus* CM1.

**Experimental factor's**	**Low (−1) level**	**High (+1) level**
pH	1	4
Temperature	25°C	45°C
NaCl	2%	8%
Bile Salt	0.5%	2%
Inoculum size	0.5%	3%
Incubation period	24 hrs	96 hrs
Ascorbic acid	0.1%	0.5%
Ammonium citrate	0.05%	0.1%
Magnesium sulfate	0.1%	0.5%
Manganese sulfate	0.1%	0.5%
Calcium carbonate	0.1%	0.5%

**Table 2:** Experimental trials of 12 runs formulated by Design Expert 12.0 software.

**Std**	**Runs**	**pH**	**Temperature (Celsius)**	**Bile salt (%)**	**NaCl %**	**Inoculum size (%)**	**Incubation period (hours)**	**Manganese sulfate (%)**	**Magnesium sulfate (%)**	**Ascorbic acid%**	**Ammonium citrate**	**Calcium carbonate**
5	1	1	25	2	2	3	96	0.1	0.5	0.5	0.05	0.1
11	2	4	25	2	8	3	24	0.1	0.1	0.5	0.1	0.5
8	3	4	45	0.5	2	0.5	96	0.1	0.5	0.5	0.1	0.5
2	4	1	45	2	2	3	96	0.5	0.1	0.1	0.1	0.5
7	5	4	25	0.5	2	3	24	0.5	0.5	0.1	0.05	0.5
12	6	1	25	0.5	2	0.5	24	0.1	0.1	0.1	0.1	0.1
6	7	1	25	0.5	8	0.5	96	0.5	0.1	0.2	0.05	0.5
10	8	1	45	2	8	0.5	24	0.1	0.5	0.1	0.05	0.5
9	9	4	45	2	2	0.5	24	0.5	0.1	0.5	0.05	0.1
4	10	1	45	0.5	8	3	24	0.5	0.5	0.5	0.1	0.1
1	11	4	45	0.5	8	3	96	0.1	0.1	0.1	0.05	0.1
3	12	4	25	2	8	0.5	96	0.5	0.5	0.1	0.1	0.1

**Table 3:** Experimental model for *Lactobacillus acidophilus* CM1 growth using the Plackett–Burman design.

**Runs**	**pH**	**Temperature (Celsius)**	**Bile salt (%)**	**NaCl (%)**	**Inoculum size (%)**	**Incubation period (hours)**	**Manganese sulfate**	**Magnesium sulfate**	**Ascorbic acid (%)**	**Ammonium citrate (%)**	**Calcium carbonate**	**Cell growth**	
												**Predicted values**	**Observed values**
1	1	25	2	2	3	96	0.1	0.5	0.5	0.05	0.1	0.706	0.721
2	4	25	2	8	3	24	0.1	0.1	0.5	0.1	0.5	0.519	0.543
3	4	45	0.5	2	0.5	96	0.1	0.5	0.5	0.1	0.5	1.009	0.995
4	1	45	2	2	3	96	0.5	0.1	0.1	0.1	0.5	0.848	0.872
5	4	25	0.5	2	3	24	0.5	0.5	0.1	0.05	0.5	1.030	1.021
6	1	25	0.5	2	0.5	24	0.1	0.1	0.1	0.1	0.1	0.940	0.912
7	1	25	0.5	8	0.5	96	0.5	0.1	0.5	0.05	0.5	0.330	0.321
8	1	45	2	8	0.5	24	0.1	0.5	0.1	0.05	0.5	0.643	0.672
9	4	45	2	2	0.5	24	0.5	0.1	0.5	0.05	0.1	1.176	1.191
10	1	45	0.5	8	3	24	0.5	0.5	0.5	0.1	0.1	0.574	0.546
11	4	45	0.5	8	3	96	0.1	0.1	0.1	0.05	0.1	0.495	0.472
12	4	25	2	8	0.5	96	0.5	0.5	0.1	0.1	0.1	0.488	0.498

**Table 4:** Analysis of P.B. design for 11 factors affecting maximum cell growth using ANOVA and regression.

**Source**	**Sum of squares**	**Df**	**Mean square**	**F** **-value**	**p** ** value**	
Model	0.7800	9	0.0867	257.42	0.0039	Significant
A—pH	0.0381	1	0.0381	113.11	0.0087	
B—temp	0.0447	1	0.0447	132.63	0.0075	
C—bile salt	0.0044	1	0.0044	13.09	0.0686	
D—NaCl	0.5896	1	0.5896	1751.39	0.0006	
E—inoculum size	0.0143	1	0.0143	42.42	0.0228	
F—incubation period	0.0843	1	0.0843	250.50	0.0040	
G—MgSO_4_.7H_2_O	0.0015	1	0.0015	4.44	0.1695	
H—MnSO_4_.4H_2_O	0.0017	1	0.0017	4.99	0.1551	
J—ascorbic acid	0.0014	1	0.0014	4.18	0.1775	
Residual	0.0007	2	0.0003			
Cor total	0.7807	11				

*Note: R* ^2^ = 99.91%; *R*^2^adjusted = 99.53%; *R*^2^predicted = 96.89%; *p* < 0.05.

Abbreviations: df = degree of freedom, MSE = mean square error, SS = sum of squares.

**Table 5:** Response surface methodology for media optimization for *Lactobacillus* yield.

**Std**	**Runs**	**PH**	**Temperature**	**NaCl%**	**Incubation period (hours)**	**Expected values**	**Observed values**
22	1	2.5	35	5	60	1.364	1.342
12	2	4	45	2	96	1.321	1.296
15	3	1	45	4	96	1.467	1.426
27	4	2.5	35	3	60	1.357	1.302
1	5	1	25	2	24	1.359	1.321
25	6	2.5	35	3	60	1.357	1.294
4	7	4	45	2	24	1.058	1.215
7	8	1	45	4	24	1.177	1.232
3	9	1	45	2	24	1.182	1.201
29	10	2.5	35	3	60	1.357	1.321
8	11	4	45	4	24	1.063	1.094
18	12	5.5	35	3	60	1.234	1.198
14	13	4	25	4	96	1.497	1.392
520	14	2.5	55	3	60	1.160	1.204
28	15	2.5	35	3	60	1.357	1.324
23	16	2.5	35	3	24	1.240	1.256
16	17	4	45	4	96	1.281	1.342
10	18	4	25	2	96	1.477	1.445
21	19	2.5	35	1	60	1.349	1.314
11	20	1	45	2	96	1.418	1.486
9	21	1	25	2	96	1.609	1.492
17	22	0.5	35	3	60	1.480	1.467
6	23	4	25	4	24	1.266	1.221
5	24	1	25	4	24	1.415	1.354
30	25	2.5	35	3	60	1.357	1.348
2	26	4	25	2	24	1.299	1.254
13	27	1	25	4	96	1.718	1.684
26	28	2.5	35	3	60	1.357	1.741
19	29	2.5	35	3	60	1.553	1.948
24	30	2.5	35	3	32	1.266	0.994

**Table 6:** Fit statistics.

Std. dev.	0.1533	*R* ^2^	0.5661
Mean	1.35	Adjusted *R*^2^	0.3377
C.V. %	11.35	Predicted *R*^2^	0.1897
		Adeq precision	7.0546

**Table 7:** Response 1: R1 [24].

**Source**	**Sum of squares**	**Df**	**Mean square**	**F** **-value**	**p** ** value**	
Model	0.5824	10	0.0582	2.48	0.0425	Significant
A—pH	0.0907	1	0.0907	3.86	0.0643	
B—temperature	0.2319	1	0.2319	9.87	0.0054	
C—NaCl	0.0003	1	0.0003	0.0147	0.9048	
D—incubation period	0.2384	1	0.2384	10.14	0.0049	
AB	0.0012	1	0.0012	0.0529	0.8206	
AC	0.0080	1	0.0080	0.3390	0.5672	
AD	0.0052	1	0.0052	0.2222	0.6428	
BC	0.0037	1	0.0037	0.1571	0.6963	
BD	0.0002	1	0.0002	0.0080	0.9295	
CD	0.0029	1	0.0029	0.1230	0.7297	
Residual	0.4464	19	0.0235			
Lack of fit	0.2954	14	0.0211	0.6985	0.7269	Not significant
Pure error	0.1510	5	0.0302			
Cor total	1.03	29				

## Data Availability

Data is available on request from the authors.
